# Supplementary material for: LRRK2-mutant microglia and neuromelanin synergize to drive dopaminergic neurodegeneration in an iPSC-based Parkinson’s disease model
Source: Commun Biol. 2025 Aug 12;8:1203. doi: 10.1038/s42003-025-08544-4 (PMC12344146; doi:10.1038/s42003-025-08544-4)
Supplement: Supplementary file 1 — Supplemental Material [file 42003_2025_8544_MOESM1_ESM.pdf]

Supplemental Information

LRRK2-mutant microglia and neuromelanin synergize to drive dopaminergic neurodegeneration in an iPSC-based Parkinson's disease model

Lucas Blasco-Agell[1,2†], Meritxell Pons-Espinal[1,2†], Veronica Testa[1,2†], Gerard Roch[3,4†], Jara Montero-Muñoz[1,2], Irene Fernandez-Carasa[1,2], Valentina Baruffi[1,2], Marta Gonzalez-Sepulveda[3,4], Yvonne Richaud-Patin[5,6], Senda Jimenez[5,6], Thais Cuadros[3,4], Joana M. Cladera-Sastre[3,4], Joan Compte[3,4], Zoe Manglano-Artuñedo[7], Salvador Ventura[7,8], Manel Juan[9], Eduardo Tolosa[10], Angel Raya[5,6,11*], Miquel Vila[3,4,11,12*], Antonella Consiglio[1*]

1. Department of Pathology and Experimental Therapeutics, Bellvitge University Hospital-IDIBELL, 08908 Hospitalet de Llobregat, Spain.
2. Institute of Biomedicine of the University of Barcelona (IBUB), Barcelona, Spain.
3. Neurodegenerative Diseases Research Group, Vall d'Hebron Research Institute (VHIR)-Network Center for Biomedical Research in Neurodegenerative Diseases (CIBERNED), Barcelona, Spain.
4. Aligning Science Across Parkinson's (ASAP) Collaborative Research Network, Chevy Chase, MD, USA.
5. Regenerative Medicine Program, Bellvitge Biomedical Research Institute (IDIBELL), and Program for Clinical Translation of Regenerative Medicine in Catalonia (P-CMRC), Hospital Duran i Reynals, Hospitalet de Llobregat, Barcelona, Spain.
6. Centre for Networked Biomedical Research on Bioengineering, Biomaterials and Nanomedicine (CIBER-BBN), Madrid, Spain.
7. Institut de Biotecnologia i de Biomedicina and Departament de Bioquímica i de Biologia Molecular, Universitat Autònoma de Barcelona, Bellaterra (Barcelona), Spain.
8. Hospital Universitari Parc Taulí, Institut d'Investigació i Innovació Parc Taulí (I3PT-CERCA), Universitat Autònoma de Barcelona, Sabadell (Spain).
9. Immunology Department–CDB, Hospital Clinic de Barcelona, Institut d'Investigacions Biomèdiques August Pi i Sunyer (IDIBAPS), University of Barcelona (UB), Barcelona, Spain.
10. Department of Neurology, Hospital Clínic de Barcelona, Institut d'Investigacions Biomèdiques August Pi i Sunyer (IDIBAPS), University of Barcelona (UB), Barcelona, Spain.
11. Catalan Institution for Research and Advanced Studies (ICREA), Barcelona, Spain.
12. Department of Biochemistry and Molecular Biology, Neuroscience Institute, Autonomous University of Barcelona, Barcelona, Spain.

[†]These authors contributed equally to this work.

*Correspondence and requests for materials should be addressed to: A.C.(email: consiglio@ub.edu) M.V. (email: miquel.vila@vhir.org) or A.R. (email: araya@idibell.cat)

Supplementary Table 1. Description of antibodies used for Immunocytochemistry.

Antibody	Host	Reactivity	Concentration	Source	Cat. number	RRID
**CX3CR1**	Rabbit	Human	1:200	Abcam	Cat# ab8020	AB_306202
**FOXA2**	Mouse	Human	1:250	Santa Cruz	Cat# sc101060	AB_1124660
**GFP**	Chicken	Human, mouse	1:250	Aves Lab	Cat# GFP-1020	AB_10000240
**IBA1**	Rabbit	Human, mouse	1:200	Wako	Cat# 019-19741	AB_839504
**IBA1**	Mouse	Human	1:200	Santa Cruz	Cat# sc-32725	AB_667733
**LMX1A**	Rabbit	Human	1:1000	Millipore	Cat# ab10533	AB_10805970
**MAP2**	Chicken	Human, mouse	1:1000	Abcam	Cat# ab5392	AB_2138153
**MAP2**	Guinea Pig	Human, mouse	1:1000	Synaptic Systems	Cat# 188 004	AB_2138181
**TH**	Rabbit	Human, rat	1:500	Sigma-Aldrich	Cat# T8700	AB_1080430
**TH**	Mouse	Human, rat	1:1000	Merck Millipore	Cat# MAB5280	AB_2201526
**TMEM119**	Mouse	Human	1:200	Biolegend	Cat# 853301	AB_2734646
**GFAP**	Guinea Pig	Human, mouse	1:1000	Synaptic Systems	Cat# 173 004	AB_10641162
**TUJ1/ TUBB3**	Mouse	Mammalian	1:500	Biolegend	Cat# 801202	AB_2313773

Supplementary Table 2. Description of antibodies employed for Flow cytometry.

Antibody	Clone	Reactivity	Concentration	Source	Cat. number	RRID
**CD11b-PE**	M1/70	Human, mouse	1:50	Biolegend®	Cat# M1/70	AB_2536062
**CD14-Beads**	-	Human	1:20	Miltenyi	Cat# 130-050-201	AB_2665482
**CD163-APC**	GHI/61.1	Human	1:50	Miltenyi	Cat# 130-128-211	AB_2904765

CD: Cluster of differentiation; PE: Picoeritrine; APC: Allophycocyanin.

Supplementary Table 3. Description of primers employed for gene expression analysis.

Gene ID	Forward primer (5' - 3')	Reverse primer (5' - 3')
C1Qa	ATGGTGACCGAGGACTTGTG	GTCCTTGATGTTTCCTGGGC
GAS6	GTAGCTTCCACTGTTCCT	GCGCACTCGTCTATGTCTT
GPR34	GAAGACAATGAGAAGTCATACC	TGTTGCTGAGAAGTTTTGTG
MerTK	CTTCTCCATGGCCACAGGTT	ATACTGAAAAGGTGGGGCGG
P2RY12	CTAAGATTCTCTGTTGTCATCTG	ACAGAGTGCTCTCTTTCACATAG
PROS1	AAGAAGCCAGGGAGGTCTTTG	ACGTGCAGCAGTGAATAACC
IL-6	AATTCGGTACATCCTCGACGG	GGTTGTTTTCTGCCAGTGCC
C3	AAAAGGGGCGCAACAAGTTC	GATGCCTTCCGGGTTCTCAA
IFNγ	CATTACCTGAAGGCCAAGGA	CTGACTATGGTCCAGGCACA
β-Actin	AGGCCAACCGCGAGAAG	ACAGCCTGGATAGCAACGTACA

Supplemental Table 4. Description of the antibodies used for Western Blot.

Antibody	Host	Reactivity	Concentration	Source	Cat. number	RRID
**PCNA**	Mouse	Human	1:3000	Sigma-Aldrich	Cat# P8825	AB_477413
**PSD-95**	Mouse	Human	1:1000	Synaptic Systems	Cat# 124 011	AB_10804286
**β-Actin**	Mouse	Human	1:2000	Affinity Biosciences	Cat# T0022	AB_2839417

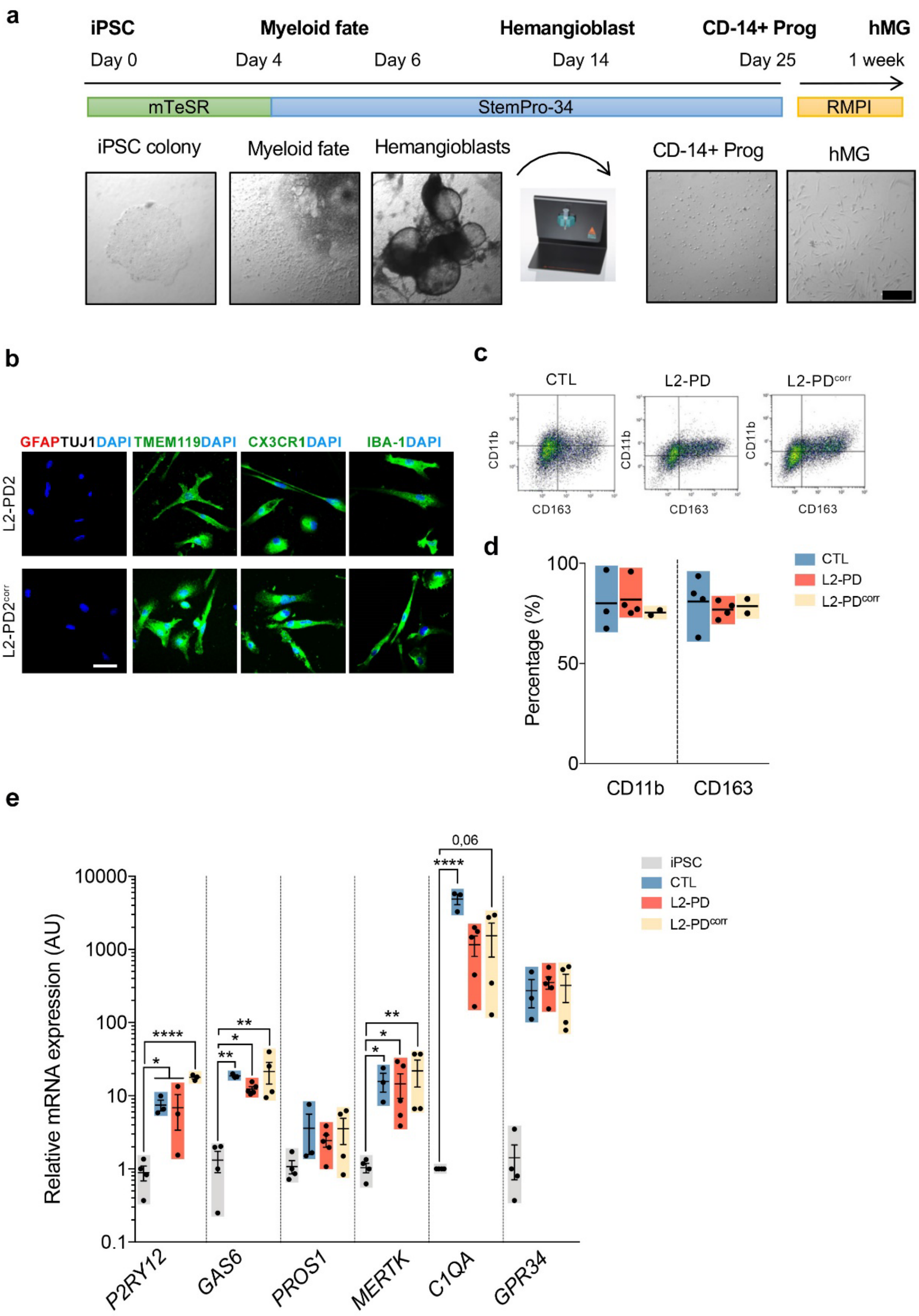

Supplementary Figure 1. Generation and characterization of hMG derived cells. a Schematic representation of the differentiation protocol to generate iPSC-derived hMG with representative bright field pictures of the process (Scale bar=100 µm). **b** Representative immunocytochemistry (ICC) images of iPSC-derived hMG after 7 days in culture from L2-PD2 (SP13) and L2-PD2^{corr} (SP13wt/wt) iPSC lines staining positive for IBA-1, CX3CR1 or TMEM-119 (green) and negative for astrocytic (GFAP) or neuronal (TUJ1) markers. Nuclei are counterstained with DAPI (blue). Scale bar=30 µm. **c-d** Representative flow cytometry plots

for CD11b and CD163 microglial surface markers from CTL (SP09), L2-PD (L2-PD2: SP13) and L2-PD^{corr} (L2-PD2^{corr}: SP13wt/wt) and its corresponding quantification. Individual data plotted, along with mean ± SEM. N=3 for CTL, N=4 for L2-PD, and N=2 L2-PD^{corr}. Intact cells were gated in a forward and side scatter (FSC/SSC) plot to exclude small debris. Gating of live cells was done using the viability dye PI. **e** Relative mRNA expression of human specific microglia genes comparing iPSCs with hMG from CTL (SP09), L2-PD (L2-PD1: SP12; L2-PD2: SP13) and L2-PD^{corr} (L2-PD1^{corr}: SP12wt/wt; L2-PD2^{corr}: SP13wt/wt). Individual data plotted, along with mean ± SEM. One-way ANOVA with Uncorrected Fisher LSD test for all comparison, except Kruskal-Wallis non parametric test with Uncorrected Dunn's test for *PROS1* and *MERKT* comparisons. N=4 for iPSC, N=3 for CTL, N=5 for L2-PD, and N=4 for L2-PD^{corr}.
*p<0.05, **p<0.01, ***p<0.001, ***p<0.0001; p-value is specified for values between 0,05 and 0,1. p-values over 0.1 (non-significant) are not shown.

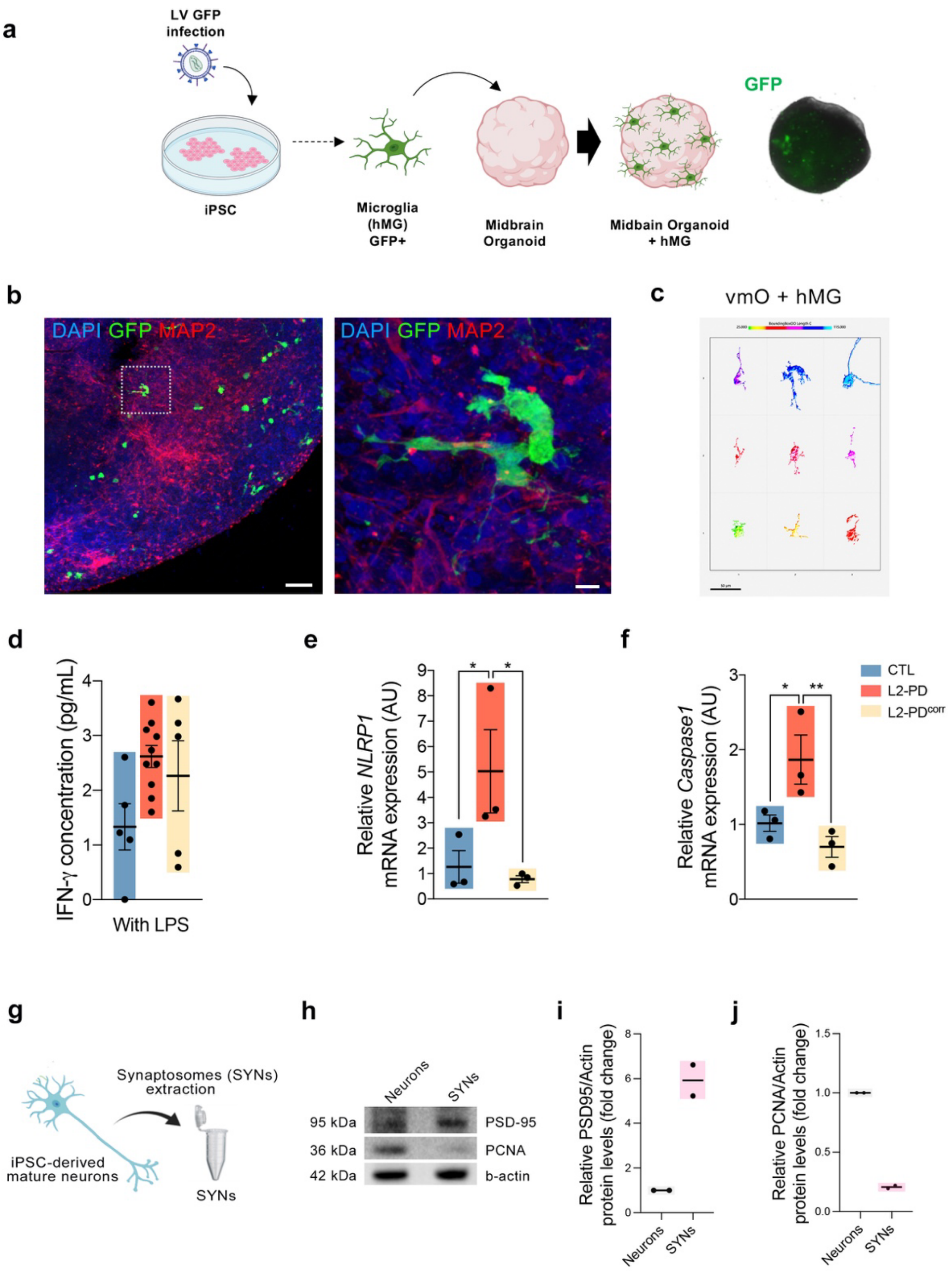

Supplementary Figure 2. 3D co-culture set up, hMG morphological analyses and functional validation. a Schematic representation of the 3D co-culture procedure. **b** Representative ICC images of iPSC-derived CTL hMG (SP09, green) inside vmO from a CTL line (SP11). Mature neurons are stained with MAP2 (red) (Scale bars=100 and 10 µm). **c** Representative IMARIS reconstructions from IN TOTO ICC of CTL hMG (SP09) showing ramified morphologies (Scale bar=50 µm). **d** Cytokine profile for IFN-γ after 24 hours of LPS stimulation in CTL (SP09), L2-PD (L2-PD1: SP12; L2-PD2: SP13) and L2-PD2^{corr} (SP13wt/wt) hMG. Individual data plotted, along with mean ± SEM. N=3 of independent experiments, each experiment containing two technical duplicates. **e-f** Relative mRNA expression of inflammasome-related genes after 24h of LPS stimulation of CTL (SP09), L2-PD (L2-PD1: SP12) and L2-PD^{corr} (L2-PD1^{corr}: SP12wt/wt) hMG. Individual data plotted, along with mean ±

SEM. One-way ANOVA with Uncorrected Fisher LSD test. N=3 of independent experiments. **g** Schematic representation of Synaptosome extraction from iPSC-derived mature neurons. **h** Representative Western blot images for Postsynaptic Density protein (PSD)-95, Proliferating Cell Nuclear Antigen (PCNA), and β-Actin protein bands from iPSC-derived Mature Neurons (Neurons) and extracted SYNs. **i-j** Quantification of PSD-95 and PCNA relative to β-Actin, represented as Fold change to Neurons. Individual data plotted, along with mean. N=2 experiments. *$p<0.05$, **$p<0.01$. p-values over 0.1 (non-significant) are not shown.

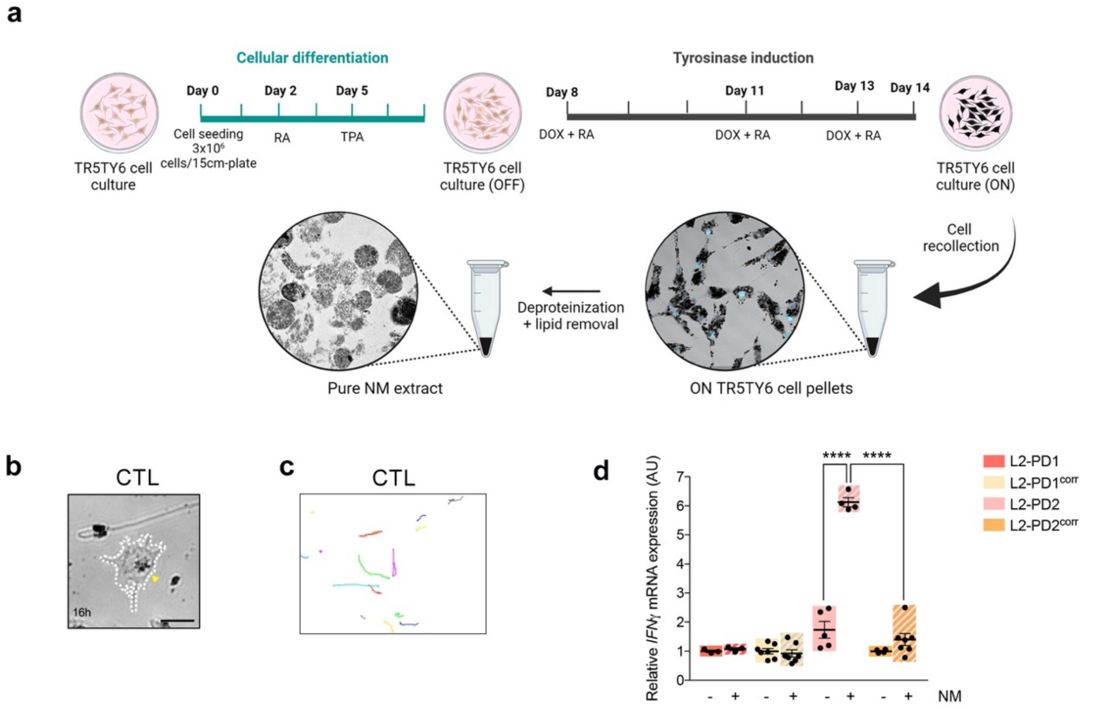

Supplementary Figure 3. NM purification from cultured TR5TY6 neuroblastoma cells. a. TR5TY6 neuroblastoma cells were differentiated for 6 days using retinoic acid (RA) and 12-O-tetradecanoylphorbol-13-acetate (TPA) and induced for human tyrosinase expression with doxycycline. Six days after induction, cells were collected and pure NM extracts were obtained after deproteinization and lipid removal. **b** Representative Bright Field images of CTL (SP09) hMG phagocyting NM particles for 16 hours (yellow arrow-heads for phagocyted particles; Scale bar=25 µm). **c** Spontaneous migration paths of CTL (SP09) hMG were tracked for 16h. The location of each cell was determined every 2 minutes and connected to depict its migration route. **d** Relative mRNA expression of IFN-γ in L2-PD1 (SP12), L2-PD1^{corr} (SP12wt/wt), L2-PD2 (SP13), and L2-PD2^{corr} (SP13wt/wt) hMG at basal conditions or under NM stimulation for 24 hours. Individual data plotted, along with mean ± SEM. One-way ANOVA with Tukey multiple comparison test. N=3 of independent experiments, each experiment containing two technical duplicates. ****p<0.0001. p-values over 0.1 (non-significant) are not shown.

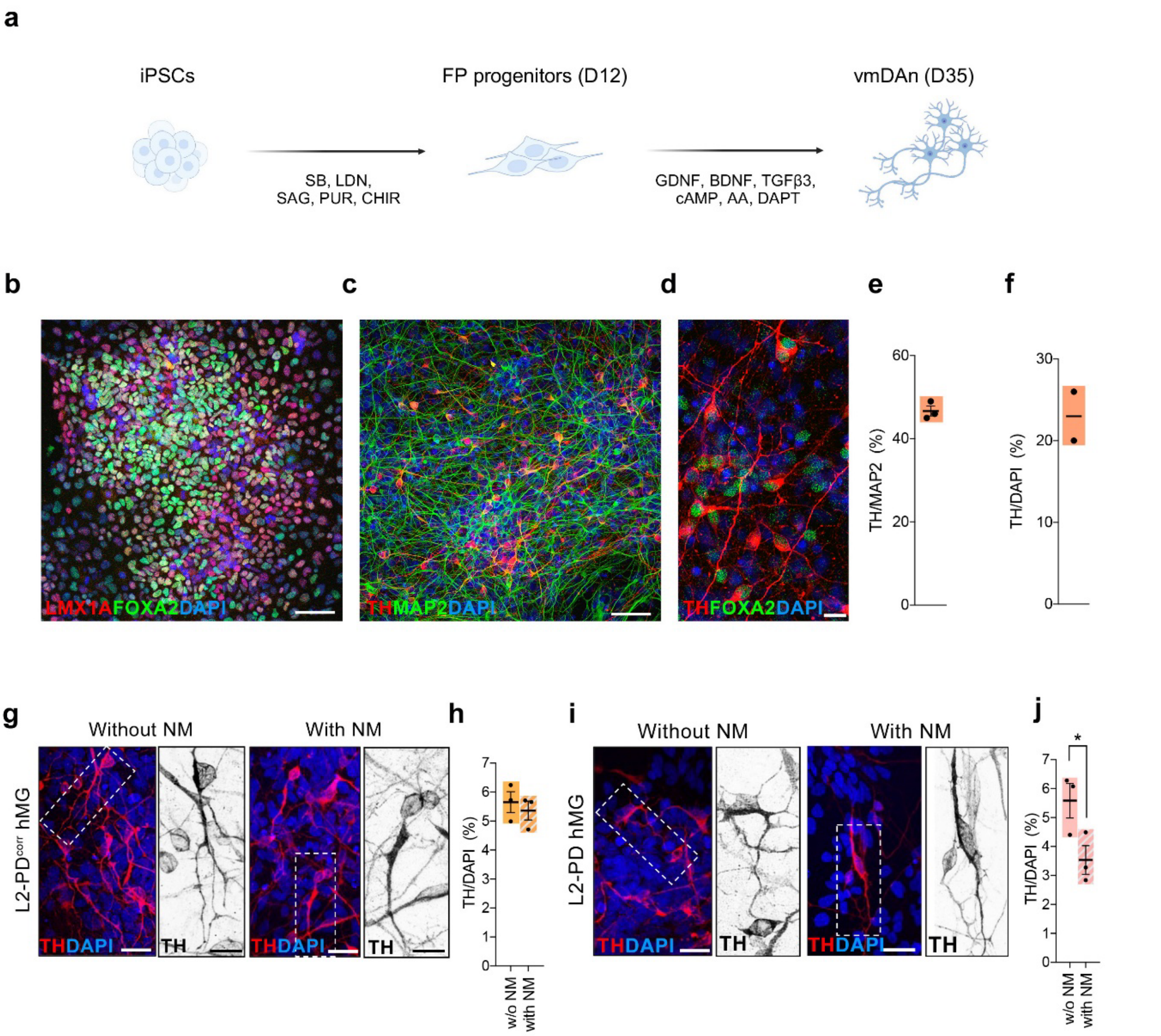

Supplementary Figure 4. vmDAn characterization and degeneration upon culture with L2-PD hMG and NM. a Schematic representation of the differentiation protocol employed to generate iPSC-derived vmDAn. **b** Representative immunocytochemistry (ICC) images of D12 CTL floor plate (FP) progenitors (SP11) staining positive for LMX1A and FOXA2. Nuclei are counterstained with DAPI (blue). Scale bar=65 µm. **c-d** Representative immunocytochemistry (ICC) images of D35 vmDAn (SP11) staining positive for TH and MAP2 (**c**) and TH and FOXA2 (d). Scale bars=50 and 13 µm. Nuclei are counterstained with DAPI (blue). **e-f** Quantification of percentage of TH/MAP2 (**e**) and TH/DAPI (**f**) in a monoculture of D35 CTL vmDAn (SP11). Individual data plotted, along with mean ± SEM. N=3 independent experiments (**e**), N=2 independent experiments (**f**). **g** Representative images of CTL TH+ vmDAn (SP11) upon culture with L2-PD2^{corr} hMG (SP13wt/wt), without or with NM (Scale bar=25 µm; 15 µm). **h** Quantification of percentage of TH+ population over the total number of DAPI+ cells in culture with L2-PD2^{corr} hMG (SP13wt/wt), without or with NM. Individual data plotted, along with mean ± SEM. Paired t test. N=3 independent experiments. **i** Representative images of CTL TH+ vmDAn (SP11) upon culture with L2-PD2 hMG (SP13), without or with NM (Scale bar=25 µm; 15 µm). **j** Quantification of percentage of TH+ population over the total number of DAPI+ cells in culture with L2-PD2 hMG (SP13), without or with NM. Individual data plotted, along with mean ± SEM. Paired t-test. N=3 independent experiments. *p<0.05. p-values over 0.1 (non-significant) are not shown.

Figure S5

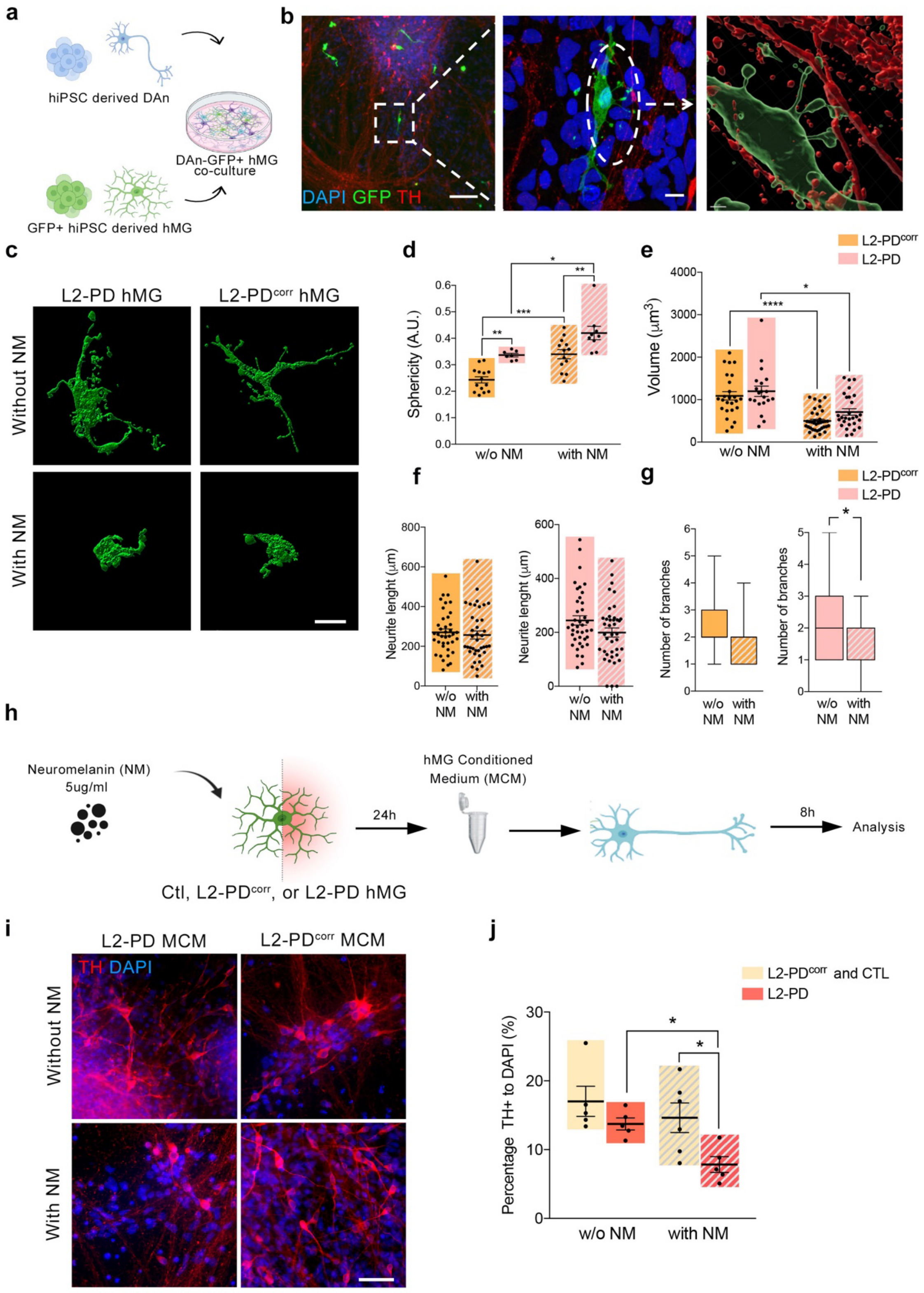

Supplementary Figure 5. Set up of iPSC-derived 2D Neuron/Microglia co-culture system. a Schematic representation of generation of a co-culture between vmDAns and GFP+ hMG. **b** Representative images of hMG (GFP+, green) in contact with CTL (SP11) TH+ vmDAns (red), with corresponding IMARIS reconstructions. Scale bar=70 µm; 7 µm; 2 µm. **c** IMARIS reconstructions from 2D Neuron/Microglia co-cultures of L2-PD (L2-PD2: SP13) and

L2-PD^{corr} (L2-PD2^{corr}: SP13wt/wt) hMG showing ramified morphologies under basal condition and amoeboid shapes after NM stimulation (Scale bar=20 µm). **d-e** Quantification of microglial sphericity (**d**; AU) and volume (**e**; µm3) with IMARIS, comparing L2-PD2^{corr} (L2-PD2^{corr}: SP13wt/wt) and L2-PD (L2-PD2: SP13) hMG, without or with NM. Individual data plotted, along with mean ± SEM. One-way ANOVA with Tukey multiple comparison test for (**d**); Kruskal-Wallis non-parametric test with Dunn multiple comparison test for (**e**). In (**d**) N=14 cells in L2-PD^{corr}, N=13 cells in the L2-PD^{corr}+NM, N=7 cells in L2-PD, N=9 in L2-PD+NM. The four conditions were measured from two independent experiments. In (**e**) N=25 cells in L2-PD^{corr}, N=36 cells in the L2-PD^{corr}+NM, N=20 cells in L2-PD, N=28 in L2-PD+NM. The four conditions were measured from two independent experiments. **f** Quantification of CTL vmDAns (SP11) neurite length (µm) upon co-culture with L2-PD^{corr} (L2-PD2^{corr}: SP13wt/wt) or L2-PD hMG (L2-PD2: SP13), under basal condition or after stimulation with NM. Individual data plotted, along with mean ± SEM. Mann-Whitney test. In (**f**) N=39 cells in L2-PD^{corr}, N=39 cells in the L2-PD^{corr}+NM, N=40 cells in L2-PD, N=40 in L2-PD+NM. The four conditions were measured from two independent experiments. **g** Quantification of the number of terminals in CTL vmDAns (SP11) upon co-culture with L2-PD^{corr} (L2-PD2^{corr}: SP13wt/wt) or L2-PD (L2-PD2: SP13) hMG, under basal condition or after stimulation with NM. Box and whiskers plot, depicting minimum and maximum value. Mann-Whitney test for L2-PD^{corr} hMG; unpaired t-test for L2-PD hMG. In (**g**) N=40 cells in L2-PD^{corr}, N=39 cells in the L2-PD^{corr}+NM, N=39 cells in L2-PD, N=39 in L2-PD+NM. The four conditions were measured from two independent experiments. **h** Schematic representation of DAn treated for 8 hours with MCM exposed to NM (5ug/ml). **i** Representative ICC images of TH+ CTL vmDAns (SP11, Red) treated with either MCM from L2-PD (L2-PD2: SP13) and L2-PD2^{corr} (L2-PD2^{corr}: SP13wt/wt) non stimulated or exposed to NM for 8 hours. Scale bar=50 µm. **j** Quantification of percentage of TH+ population over DAPI upon treatment with MCM from control or L2-PD (L2-PD1:SP12 and L2-PD2: SP13) hMG, non-stimulated or exposed to NM for 8 hours. CTL (SP09) and L2-PD^{corr} (SP13wt/wt) hMG have been pooled together and considered as control group. Individual data plotted, along with mean ± SEM. Kruskal-Wallis non-parametric test with Uncorrected Dunn's test. For L2-PD^{corr} and CTL lines at basal conditions N=5 measurements coming from two independent experiments. For L2-PD at basal conditions and with NM N=5 measurements coming from two independent experiments. For L2-PD^{corr} and CTL with NM N=6 measurements coming from two independent experiments.

*p<0.05, **p<0.01, ***p<0.001, ***p<0.0001. p-values over 0.1 (non-significant) are not shown.

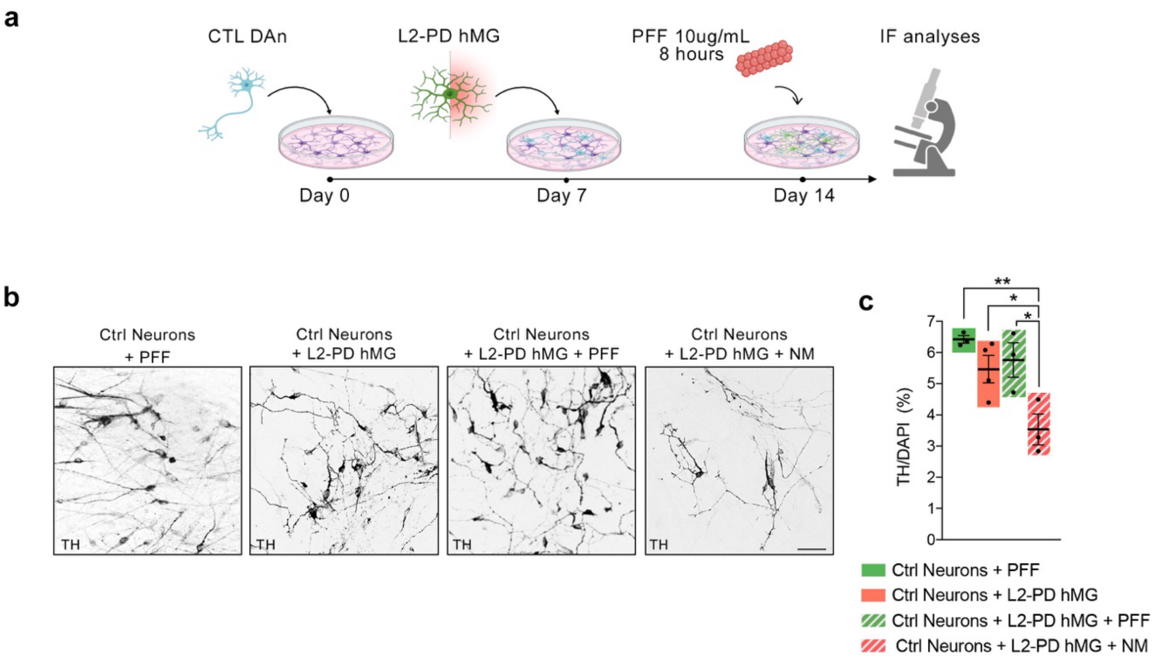

Supplementary Figure 6. L2-PD hMG exposed to α-synuclein pre-formed fibrils (PFF) does not trigger DAn degeneration in an iPSC-derived 2D neuron/hMG co-culture system. a Schematic representation of the co-culture system and PFF stimulation (10ug/ml). **b** Representative images of TH+ (black) CTRL (SP11) vmDAns treated with PFF, or in co-culture with L2-PD (L2-PD2: SP13) hMG treated with either PFF or NM (5ug/ml) (Scale bar=50 µm). **c** Quantification of percentage of TH+ population over the total number of DAPI+ cells. Individual data plotted, along with mean ± SEM. One-way ANOVA with Tukey multiple comparison test. N=4 for Ctrl Neurons + L2-PD hMG and N=3 for the other three conditions. *p<0.05, **p<0.01. p-values over 0.1 (non-significant) are not shown.
