# Supplementary material for: LRRK2-mutant microglia and neuromelanin synergize to drive dopaminergic neurodegeneration in an iPSC-based Parkinson’s disease model
Source: Commun Biol. 2025 Aug 12;8:1203. doi: 10.1038/s42003-025-08544-4 (PMC12344146; doi:10.1038/s42003-025-08544-4)
Supplement: Supplementary file 2 — Description of Additional Supplementary Files [file 42003_2025_8544_MOESM2_ESM.docx]

**Description of Additional Supplementary Files**

File name: Supplementary Data 1

Description: Source data behind the graphs in the paper.

File name: Supplementary Movie 1

Description: Example of a control (SP09) microglia phagocyting NM particles. Total video duration of 5 hours, with one image taken every three minutes (8 fps, 20X objective).
