# Supplementary material for: LRRK2-mutant microglia and neuromelanin synergize to drive dopaminergic neurodegeneration in an iPSC-based Parkinson’s disease model
Source: Commun Biol. 2025 Aug 12;8:1203. doi: 10.1038/s42003-025-08544-4 (PMC12344146; doi:10.1038/s42003-025-08544-4)
Supplement: Supplementary file 5 — Reporting Summary [file 42003_2025_8544_MOESM5_ESM.pdf]

Corresponding author(s): Antonella Consiglio

Last updated by author(s): 06/12/2025

Reporting Summary

Nature Portfolio wishes to improve the reproducibility of the work that we publish. This form provides structure for consistency and transparency in reporting. For further information on Nature Portfolio policies, see our Editorial Policies and the Editorial Policy Checklist.

Statistics

For all statistical analyses, confirm that the following items are present in the figure legend, table legend, main text, or Methods section.

n/a	Confirmed	
☐	☒	The exact sample size (*n*) for each experimental group/condition, given as a discrete number and unit of measurement
☐	☒	A statement on whether measurements were taken from distinct samples or whether the same sample was measured repeatedly
☐	☒	The statistical test(s) used AND whether they are one- or two-sided<br>*Only common tests should be described solely by name; describe more complex techniques in the Methods section.*
☒	☐	A description of all covariates tested
☐	☒	A description of any assumptions or corrections, such as tests of normality and adjustment for multiple comparisons
☐	☒	A full description of the statistical parameters including central tendency (e.g. means) or other basic estimates (e.g. regression coefficient) AND variation (e.g. standard deviation) or associated estimates of uncertainty (e.g. confidence intervals)
☐	☒	For null hypothesis testing, the test statistic (e.g. *F*, *t*, *r*) with confidence intervals, effect sizes, degrees of freedom and *P* value noted<br>*Give P values as exact values whenever suitable.*
☒	☐	For Bayesian analysis, information on the choice of priors and Markov chain Monte Carlo settings
☒	☐	For hierarchical and complex designs, identification of the appropriate level for tests and full reporting of outcomes
☒	☐	Estimates of effect sizes (e.g. Cohen's *d*, Pearson's *r*), indicating how they were calculated

Our web collection on statistics for biologists contains articles on many of the points above.

Software and code

Policy information about availability of computer code

Data collection

Images were acquired using Zeiss AXIOIMAGER Z1 with an ApoTome (Zeiss Microscopy) microscope using a Cascade CCD camera (Photometrics). For confocal images and time-lapse videos, a Zeiss AXIOIMAGER Z1 with an SPE confocal system or a Zeiss LSM 880 were employed (Zeiss Microscopy). Image acquisition was realized with ZEN pro software (Zeiss Microscopy). Image acquisition for postmortem brain slices was performed using an Olympus Slideview VS200 slide scanner. Flow cytometry measurement was done with the Gallios cytometer (Beckman Coulter Life Sciences) with the specific filters for each fluorochrome. RNA expression analysis was run in a 7900HT Fast Real-Time PCR System with 384-well Block Module (Applied Biosystems™). The EMD MILLIPLEX® MAP Human Cytokine/Chemokine/Growth Factor Panel A – Immunology Multiplex Assay (Merck Millipore) was run on a multiplex system Luminex® 200TM (Invitrogen™). Western blot images were obtained using a ChemiDoc System (Bio-Rad Laboratories). ROS measurement was acquired measuring the fluorescence at 485nm/530nm with a FLUOstar Omega microplate reader (BMG LABTECH) .

Data analysis	Images were analysed using ImageJ (NIH), Photoshop® (Adobe) and IMARIS (Bitplane copyright). Scanned postmortem brain sections were analysed with a specifically trained A-powered algorithm using the Olympus V200 Desktop 3.3 software to quantify IBA-1+ cells and their morphology. FACS analyses were done with Kaluza software (Beckman Coulter Life Sciences). Multiplex cytokine assay was analysed with the xPONENT® software (Luminex®).<br>Statistical analyses were performed using GraphPad Prism version 7.0a for MacOSX, (GraphPad Software, Boston, Massachusetts USA, www.graphpad.com). Outlier values were determined using ROUT test with Q set to 1%. Data normality was assessed with Shapiro-Wilk test for n<10 or by Kolmogorov-Smirnov or D'agostino-Pearson test for n>10. For normally distributed data, pairwise comparisons were done using two-tailed t-test. For data comprising more than two groups, one-way ANOVA was used followed by a post-hoc test, as specified in figure legends. For data departing from normality, Mann-Whitney test was used when two groups were compared, or Kruskal-Wallis followed by a post-hoc test as specified in figure legends when comparing more than two groups. p_values lower than 0.05 were considered significant.

For manuscripts utilizing custom algorithms or software that are central to the research but not yet described in published literature, software must be made available to editors and reviewers. We strongly encourage code deposition in a community repository (e.g. GitHub). See the Nature Portfolio guidelines for submitting code & software for further information.

Data

Policy information about availability of data

All manuscripts must include a data availability statement. This statement should provide the following information, where applicable:

- Accession codes, unique identifiers, or web links for publicly available datasets
- A description of any restrictions on data availability
- For clinical datasets or third party data, please ensure that the statement adheres to our policy

All data supporting the findings of this study are available within the paper and its Supplementary Information.

Research involving human participants, their data, or biological material

Policy information about studies with human participants or human data. See also policy information about sex, gender (identity/presentation), and sexual orientation and race, ethnicity and racism.

Reporting on sex and gender	Gender of Human postmortem samples is specified in Table 2 (Human Sample information).
Reporting on race, ethnicity, or other socially relevant groupings	Human postmortem brain samples are carefully described in Table 2 (Human Sample Information). Case status reporting Control or L2-PD cases is properly described. For L2-PD cases, the disease duration (years) is also specified.
Population characteristics	The population characteristics on human research participants is provided in Methods section: Human post-mortem brain tissue and Table 2 (Human Sample information).
Recruitment	Participants were recruited at the Neurological Tissue Bank of the Biobanc-Hospital Clinic-IDIBAPS (Barcelona, Spain).
Ethics oversight	All procedures were conducted in accordance with guidelines established by the BPC (CPMP/ICH/135/95) and the Spanish regulation (223/2004) and approved by the Vall d'Hebron Research Institute (VHIR) Ethical Clinical Investigation Committee [PR(AG)370/2014].

Note that full information on the approval of the study protocol must also be provided in the manuscript.

Field-specific reporting

Please select the one below that is the best fit for your research. If you are not sure, read the appropriate sections before making your selection.

☒ Life sciences ☐ Behavioural & social sciences ☐ Ecological, evolutionary & environmental sciences

For a reference copy of the document with all sections, see nature.com/documents/nr-reporting-summary-flat.pdf

Life sciences study design

All studies must disclose on these points even when the disclosure is negative.

Sample size	Sample size was chosen based on previous experience and standards in the field.
Data exclusions	Outlier values were determined and excluded using ROUT test with Q set to 1% (GraphPad Prism version 7.0 for MacOSX).
Replication	All experimental findings were reliably reproduced. Each experiment was repeated independently and values showed correspond to the mean value and SEM as indicated in Statistical Analysis Methods.
Randomization	Cells were randomly distributed in the cell culture plate and assigned to experimental groups.
Blinding	Investigators were blinded to the groups and samples.

Reporting for specific materials, systems and methods

We require information from authors about some types of materials, experimental systems and methods used in many studies. Here, indicate whether each material, system or method listed is relevant to your study. If you are not sure if a list item applies to your research, read the appropriate section before selecting a response.

Materials & experimental systems

n/a	Involved in the study	
☐	☒	Antibodies
☐	☒	Eukaryotic cell lines
☒	☐	Palaeontology and archaeology
☒	☐	Animals and other organisms
☒	☐	Clinical data
☒	☐	Dual use research of concern
☒	☐	Plants

Methods

n/a	Involved in the study	
☒	☐	ChIP-seq
☐	☒	Flow cytometry
☒	☐	MRI-based neuroimaging

Antibodies

Antibodies used	Antibodies providers and additional information are given in the Supplemental Information and Methods section of the main manuscript. Supplementary Table 1, 2, and 4 describe the antibodies used for immunocytochemistry, Flow cytometry assays, and Western Blot, respectively.
Validation	All the antibodies used in this study have been previously reported and are commercially available. All of them have an online data sheet reporting the validity for the species and application tested.

Eukaryotic cell lines

Policy information about cell lines and Sex and Gender in Research

Cell line source(s)	The parental iPSC lines used in this study were previously generated and fully characterized (Sanchez-Danes, 2012). The isogenic controls (L2-PDcorr: SP13 wt/wt and SP12 wt/wt), were previously obtained by correcting the LRRK2 mutation in the SP13 iPSC line (Di Domenico, 2019) and SP12 iPSC line (Carola, 2021).
Authentication	The identity of iPSC cell lines and its capacity for differentiation was routinely confirmed in the lab.
Mycoplasma contamination	All cell lines were routinely assessed for mycoplasma and tested negative.
Commonly misidentified lines (See ICLAC register)	No commonly misidentified cell lines were used.

Plants

Seed stocks	*Report on the source of all seed stocks or other plant material used. If applicable, state the seed stock centre and catalogue number. If plant specimens were collected from the field, describe the collection location, date and sampling procedures.*
Novel plant genotypes	*Describe the methods by which all novel plant genotypes were produced. This includes those generated by transgenic approaches, gene editing, chemical/radiation-based mutagenesis and hybridization. For transgenic lines, describe the transformation method, the number of independent lines analyzed and the generation upon which experiments were performed. For gene-edited lines, describe the editor used, the endogenous sequence targeted for editing, the targeting guide RNA sequence (if applicable) and how the editor was applied.*
Authentication	*Describe any authentication procedures for each seed stock used or novel genotype generated. Describe any experiments used to assess the effect of a mutation and, where applicable, how potential secondary effects (e.g. second site T-DNA insertions, mosiacism, off-target gene editing) were examined.*

Flow Cytometry

Plots

Confirm that:

☒ The axis labels state the marker and fluorochrome used (e.g. CD4-FITC).

☒ The axis scales are clearly visible. Include numbers along axes only for bottom left plot of group (a 'group' is an analysis of identical markers).

☒ All plots are contour plots with outliers or pseudocolor plots.

☒ A numerical value for number of cells or percentage (with statistics) is provided.

Methodology

Sample preparation	Cells were detached, centrifuged at 300xg for 10 minutes and pellet was incubated with 1:50 conjugated antibody in MACS buffer for 15 minutes protected from the light (see Supplementary Table 2). After that, cold MACS buffer was added, and cells were centrifuged at 300xg for 10 minutes. Cell pellet was resuspended in cold MACS buffer and incubated with 1:500 of Propidium iodide (Pi) at Room temperature (RT) for 5 minutes to determine cellular viability of the culture. Unstained cells were used as negative controls.
Instrument	Flow Cytometry measurement was done with the Gallios cytometer (Beckman Coulter Life Sciences) with the specific filters for each fluorochrome.
Software	Analyzes of staining percentage and fluorescence intensities were done with Kaluza software (Beckman Coulter Life Sciences).
Cell population abundance	Fluorescence data acquisition for positive or negative cells was performed by recording 10000 events from an homogeneous cell population.
Gating strategy	Intact cells were gated in an forward and side scatter (FSC/SSC) plot to exclude small debris. Gating of live cells was done using the viability dye PI.

☐ Tick this box to confirm that a figure exemplifying the gating strategy is provided in the Supplementary Information.
